# Identification of Suppressors of *top-2* Embryonic Lethality in *Caenorhabditis elegans*

**DOI:** 10.1534/g3.119.400927

**Published:** 2020-02-21

**Authors:** Nirajan Bhandari, Christine Rourke, Thomas Wilmoth, Alekya Bheemreddy, David Schulman, Dina Collins, Harold E. Smith, Andy Golden, Aimee Jaramillo-Lambert

**Affiliations:** * Department of Biological Sciences, University of Delaware, Newark, Delaware 19701; † National Institute of Diabetes and Digestive and Kidney Diseases, National Institutes of Health, Bethesda, Maryland 20892

**Keywords:** *top-2*, *C. elegans*, meiosis, topoisomerase II

## Abstract

Topoisomerase II is an enzyme with important roles in chromosome biology. This enzyme relieves supercoiling and DNA and RNA entanglements generated during mitosis. Recent studies have demonstrated that Topoisomerase II is also involved in the segregation of homologous chromosomes during the first meiotic division. However, the function and regulation of Topoisomerase II in meiosis has not been fully elucidated. Here, we conducted a genetic suppressor screen in *Caenorhabditis elegans* to identify putative genes that interact with *topoisomerase II* during meiosis. Using a temperature-sensitive allele of *topoisomerase II*, *top-2**(**it7ts**)*, we identified eleven suppressors of *top-2*-induced embryonic lethality. We used whole-genome sequencing and a combination of RNAi and CRISPR/Cas9 genome editing to identify and validate the responsible suppressor mutations. We found both recessive and dominant suppressing mutations that include one intragenic and 10 extragenic loci. The extragenic suppressors consist of a known Topoisomerase II-interacting protein and two novel interactors. We anticipate that further analysis of these suppressing mutations will provide new insights into the function of Topoisomerase II during meiosis.

Type II topoisomerases are highly conserved, ATP-dependent enzymes that alleviate DNA entanglements by passing one DNA strand through a transient break they create in a separate strand (Nitiss 2009). In this way, type II topoisomerases (Topo II) solve a variety of topological problems that arise during mitotic processes such as DNA replication, transcription, sister chromatid separation, and recombination (Nitiss 2009). In contrast to mitosis, meiosis has two nuclear divisions (Meiosis I and Meiosis II). In Meiosis I, homologous chromosomes segregate, which requires pairing, synapsis, and recombination. In Meiosis II, sister chromatids segregate from each other similar to mitosis. Studies in mammals, yeast, *Drosophila*, and our recent studies in *C. elegans* demonstrate that Topo II plays a role in homologous chromosome segregation at Meiosis I (Rose and Baillie 1980; Hartsuiker *et al.* 1998; Li *et al.* 2013; Hughes and Hawley 2014; Mengoli *et al.* 2014; Jaramillo-Lambert *et al.* 2016). We previously identified a temperature-sensitive (ts) allele of the single *C. elegans* Topo II homolog, *top-2**(**it7ts**)*. At the nonpermissive temperature (24°), *top-2**(**it7ts**)* disrupts the segregation of homologous chromosomes during the first meiotic division resulting in chromatin bridges during spermatogenesis. Chromatin bridging leads to a failure in the proper segregation of homologous chromosomes during Meiosis I resulting in anucleate sperm and embryonic lethality after fertilization (Jaramillo-Lambert *et al.* 2016). Interestingly, this allele does not display any chromosome segregation defects during oocyte meiosis. However, where Topo II functions within the genetic pathway of meiosis, as well as its meiosis interacting partners, have not been elucidated.

We sought to identify putative genes that interact with *top-2* during meiosis through the application of a genetic suppressor screen. Genetic screens have the advantage of an unbiased approach in the identification of genes essential for a given pathway (Hodgkin 2005). In addition, these types of screens often lead to the identification of genes that were not previously believed to function in the pathway of interest. Compromised or reduction-of-function alleles rather than null alleles are ideal for suppressor screens because they increase the likelihood that a second-site, extragenic mutation will ameliorate the phenotype of a given process (Jorgensen and Mango 2002). For these reasons we used the *C. elegans* temperature-sensitive mutant strain, *top-2**(**it7ts**)*, for our screen. With this approach, we report the identification and characterization of 11 suppressors of *top-2**(**it7ts**)* embryonic lethality. The suppressors include both dominant and recessive alleles and are found both in intragenic and extragenic loci. We used one-step whole-genome sequencing (WGS) and single-nucleotide polymorphism (SNP) mapping to identify the responsible mutations for all 11 of the suppressor lines. This approach identified genes/proteins that have been previously shown to interact with Topo II in other systems as well as two novel interactors.

## Materials And Methods

### Nematode strains

*C. elegans* strains (Table S1) were maintained using standard conditions (Brenner 1973).

### EMS suppressor screen

*unc-4**(**e120**) **top-2**(**it7ts**)* L4 hermaphrodites were soaked in 48 mM EMS for 4 h at 15°, washed three times in M9, placed on a fresh 100 mm MYOB plate with OP50 and allowed to recover for 4 h at 15°. 25 L4s (P0s) were picked to each of 40 100 mm MYOB plates with OP50 and incubated at 15°. ∼60 h later the P0s were removed from the plates. Gravid F1 adult progeny (84,720 haploid F1 genomes) were subjected to hypochlorite treatment to isolate F2 embryos. The F2 progeny were grown at 24° and their progeny were screened for viable animals. Eleven stable lines from independent P0s were identified.

### Whole-genome sequencing and SNP mapping

The molecular identification of the *top-2**(**it7ts**)* suppressors were determined by Hawaiian single-nucleotide polymorphism (SNP) mapping and whole-genome sequencing (WGS) (Doitsidou *et al.* 2010). All suppressors were crossed with Hawaiian (CB4856) males and screened for F2 suppressors as in (Jaramillo-Lambert *et al.* 2015). For suppressors *ude2*, *ude3*, *ude4*, *ude5*, *ude6*, *ude7*, and *ude24* libraries were prepared and sequenced as in (Jaramillo-Lambert *et al.* 2015). Libraries and sequencing for suppressors *ude13*, *ude14*, *ude15*, and *ude16* were prepared and sequenced by the University of Delaware DNA Sequencing and Genotyping Center. Suppressing mutations were identified by a pipeline of BBMAP (Bushnell), SAMtools (Li *et al.* 2009), FreeBayes (Garrison and Marth), and ANNOVAR (Wang *et al.* 2010); see (Smith and Yun 2017) for detailed command-line instructions.

### Embryonic viability assays

Individual L4 hermaphrodite larvae were placed on a single 35 mm MYOB plate spotted with OP50 or *E. coli* HT115[DE3] containing plasmids for RNAi knockdown. Each hermaphrodite was allowed to lay embryos at 24° for 24 h. Every 24 h period, adult worms were transferred to fresh plates until no additional embryos were produced. Percent hatching was calculated as the number of hatched larvae divided by the total number of embryos laid.

### Genetic analyses

#### Dominance/recessive tests:

To determine whether each *top-2**(**it7ts**)* suppressor was either a dominant or recessive mutation, each suppressor line [*unc-4**(**e120**) **top-2**(**it7ts**)*; *sup*] was crossed with *top-2**(**it7ts**)*; *him-8**(**e1489**)* males at 15°. Non-unc L4 cross progeny [*unc-4**(**e120**) **top-2**(**it7ts**)/**unc-4**(+) **top-2**(**it7ts**)*; *sup/+*] were picked to individual plates for embryonic viability assays at 24°. Nonviable progeny (<10% viable) indicated that the suppressing mutation was recessive and viable progeny indicated that the suppressing mutation was dominant (>50% viable) or semi-dominant (10–49% viable).

#### Linkage analysis:

To distinguish between linked and unlinked suppressor mutations, F1 progeny [*unc-4**(**e120**) **top-2**(**it7ts**)/**unc-4**(+) **top-2**(**it7ts**)*; *sup/+*] from the above cross were picked to new plates and allowed to produce self-progeny at 15°. 16-48 Unc F2 progeny [either *unc-4**(**e120**) **top-2**(**it7ts**)/ **unc-4**(**e120**) **top-2**(**it7ts**)*; *+/+*, *unc-4**(**e120**) **top-2**(**it7ts**)/ **unc-4**(**e120**) **top-2**(**it7ts**)*; *sup/+*, *or **unc-4**(**e120**) **top-2**(**it7ts**)/ **unc-4**(**e120**) **top-2**(**it7ts**)*; *sup/sup*] were picked to individual plates for embryonic viability assays. Dominant suppressors were determined to be unlinked from *top-2* if 3/4 of F2 animals had viable progeny. Recessive suppressors were determined to be unlinked from *top-2* if 1/4 of F2 animals had viable progeny. Suppressors were determined to be linked if all F2 animals had viable progeny. These results were confirmed by WGS/SNP analysis.

### RNAi

RNA was introduced into worms using the feeding method of (Timmons *et al.* 2001). RNAi clones were obtained from the Ahringer library (*smd-1*, *nurf-1*) or the OpenBiosytems library (*viln-1*, *mep-1*, *tdpt-1*). The *nlp-38* clone was made by amplifying a 540 bp DNA fragment using primers CGTGTGTTGGGATGGAATAAGG and CTTCCCCAAAGACCATTGGC. The *top-3* clone was made by amplifying a 481 bp DNA fragment using primers CAAGTGATCAGCTATGGC and GCACCTCATTAGCAAAATCTCCCC. These fragments were inserted into pDEST-L4440 using Gateway technology (Invitrogen) and transformed into *E. coli* HT115[DE3].

### CRISPR/Cas9-mediated genome editing

CRISPR-mediated genome editing via the clone-free method (Paix *et al.* 2015) with *dpy-10* as a co-CRISPR marker (Arribere *et al.* 2014) was performed to recreate the *ude5* and *ude6* candidate mutations in KK381 [*unc-4**(**e120**) **top-2**(**it7ts**)*]. All injections were done using an injection mix of Cas9 protein (25 μg), *dpy-10* crRNA (3.2 μg, Dharmacon, GE Life Sciences, Pittsburgh, PA), *dpy-10**(**cn64**)* repair oligonucleotide (0.2 μg), universal tracrRNA (20 μg, Dharmacon), an allele-specific crRNA (8 μg) and an allele-specific repair oligonucleotide (3.8 μg). To recreate the *top-2**(**ude6**)* allele, which changes Asp809 to Asn, a crRNA targeting the sequence **AATCTCGCTCAAGATTACGTTGG and a repair template (GGAGAACAGTCGCTTATGGGAACAATTGTGAATCT**AGCTCA*G***A**ATTA*T*GT*C*GGCTCCAACAACATCAACCTGCTTCTTCCAATCGGAC) with sequence to edit codon 809 (bold), mutations to prevent additional cleavage by Cas9 (italics), and an *Alu*I recognition site (underlined) for genotyping were used. *tdpt-1**(**ude5**)*, which changes Gly270 to Asp, was recreated using a crRNA targeting the sequence CCGGGCGCCCTCGTCTTTTT and a repair template (GTCCGTGAAATCATCGCTCAAAACCCGGGCGCCCT*G*GT*G*TT*C*TT*T*GGCG**A**CGATTTAAACTTACGAGACGAGGAGGTCAGCCGTGTGCCTGACG) with sequence to edit codon 270 (bold), mutations to prevent additional cleavage by Cas9 (italics), and a *Dra*I recognition site (underlined) for genotyping.

### DAPI staining

Hermaphrodites were synchronized by picking L4s and incubating at either 15° or 24° for 16-24 h. Gonads were dissected on a coverslip in 30 μl 1X Egg buffer (118 mM NaCl, 48 mM KCl, 2 mM CaCl_2_, 2 mM MgCl_2_, 25 mM Hepes, pH 7.4) plus 0.1% Tween 20. Following dissection and extrusion of the gonads, 15 μl of the Egg buffer was removed and discarded. 15 μl of 2% paraformaldehyde solution in 1X Egg buffer plus 0.1% Tween 20 (16% paraformaldehyde, Electron Microscopy Sciences, Hatfield, PA) was then added to the slide. This was followed by removal of 15 μl of the paraformaldehyde/Egg/Tween solution and a Superfrost Plus microscope slide (Fisher Scientific) was placed on top. The samples were allowed to fix for 5 min and then frozen on dry ice. The slides were then placed in -20° methanol for 1 min and washed three times in PBST (1X PBS, 0.1% Tween 20) for five min each. Slides were incubated in the dark with DAPI (2 μg/ml) for 5 min followed by a final wash in PBST for 5 min. Slides were mounted with Vectashield (Vector Laboratories) and the edges sealed with clear nail polish.

### Image collection and processing

Z-stack images of post-meiotic sperm were obtained using a Zeiss LSM780 confocal microscope (Carl Zeiss, Inc., Gottingen, Germany). For each sample, the appropriate range of focal planes for z-stack projections was chosen with a constant slicing of 0.2 μm. Image processing and analysis was done via Fiji Is Just ImageJ [Fiji, (Schindelin *et al.* 2012)]. Although images were obtained using identical imaging parameters, brightness and contrast were adjusted to allow better visualization.

### Antibody production

*C. elegans*-specific anti-TOP-2 antibodies were produced by injecting rabbits with the synthetic peptide (CQRDPKMNTIKITINKEKNE) (YenZym Antibodies, LLC, San Francisco, CA). Polyclonal antibodies were purified by antigen-specific affinity purification (YenZym Antibodies, LLC, San Francisco, CA). Specificity of the antibody was validated by Western blot and reduction of protein in the *top-2**(**ok1930**)* deletion line and worms depleted of TOP-2 through RNAi (Figure S2).

### Preparation of whole-worm protein lysates and Western blotting

Seven to ten L4 worms from each strain were picked to six 60 mm plates and grown until a majority of their progeny had reached L4 stage. Worms were then shifted to 24° for 24 h. Following incubation, plates were washed with M9 buffer and placed into a 1.5 mL tube. Worms were washed three times with M9 and allowed to settle by gravity forming a worm pellet. Then the M9 was removed and an equivalent volume of 3X-SDS Sample buffer (3% SDS, 30% glycerol, 188 mM Tris, 0.01% Bromophenol blue, and 15% β-Mercaptoethanol) was added to the pellet. Next, samples were frozen at -80° for 15 min, boiled at 95° for 10 min, and vortexed for 10 min. Samples were then centrifuged at 14,000 RPM for 30 min at 4°. The resulting supernatants were extracted and placed in fresh tubes. If not immediately used, lysates were stored at -20°. 20 µL of each sample were loaded onto a 4–15% precast gel (Bio-Rad Laboratories, Hercules, CA). The gel was run at 200V and then was transferred onto a 0.45 µm nitrocellulose membrane (Bio-Rad Laboratories, Hercules, CA). The membranes were blocked using 5% milk (in TBST) for 1 h. The membranes were blotted for TOP-2 using anti-TOP-2 primary antibody (1:750 dilution in 5% milk/TBST) and for anti-alpha tubulin using DM1α (Sigma), (1:5000 dilution in milk/TBST), for 16-20 h at 4°. Membranes were washed with TBST. The membranes were incubated in secondary antibodies (anti-rabbit HRP-conjugated and anti-mouse HRP-conjugated antibodies, Life Technologies, 1:10,000 dilution in TBST), at room temperature for 1 h. Proteins were detected with Clarity MAX ECL Western blotting substrate and the blots were imaged with a ChemiDoc Imaging System (Bio-Rad Laboratories, Hercules, CA).

### Data availability

All strains and antibodies are available upon request. The authors state that all data are represented within the article. Figure S1 demonstrates that the RNAi knockdown of suppressor candidates do not cause embryonic lethality in a wild-type genetic background. Figure S2 shows the specificity of the *C. elegans* anti-TOP-2 antibody. Table S1 is a list of the *C. elegans* strains used in this study. Table S2 shows that RNAi of suppressor candidates in the suppressor background does not reverse suppression. Supplemental material available at figshare: https://doi.org/10.25387/g3.11858025.

## Results And Discussion

### Isolation and mapping of suppressors of top-2(it7ts) embryonic lethality

**Figure 1:**
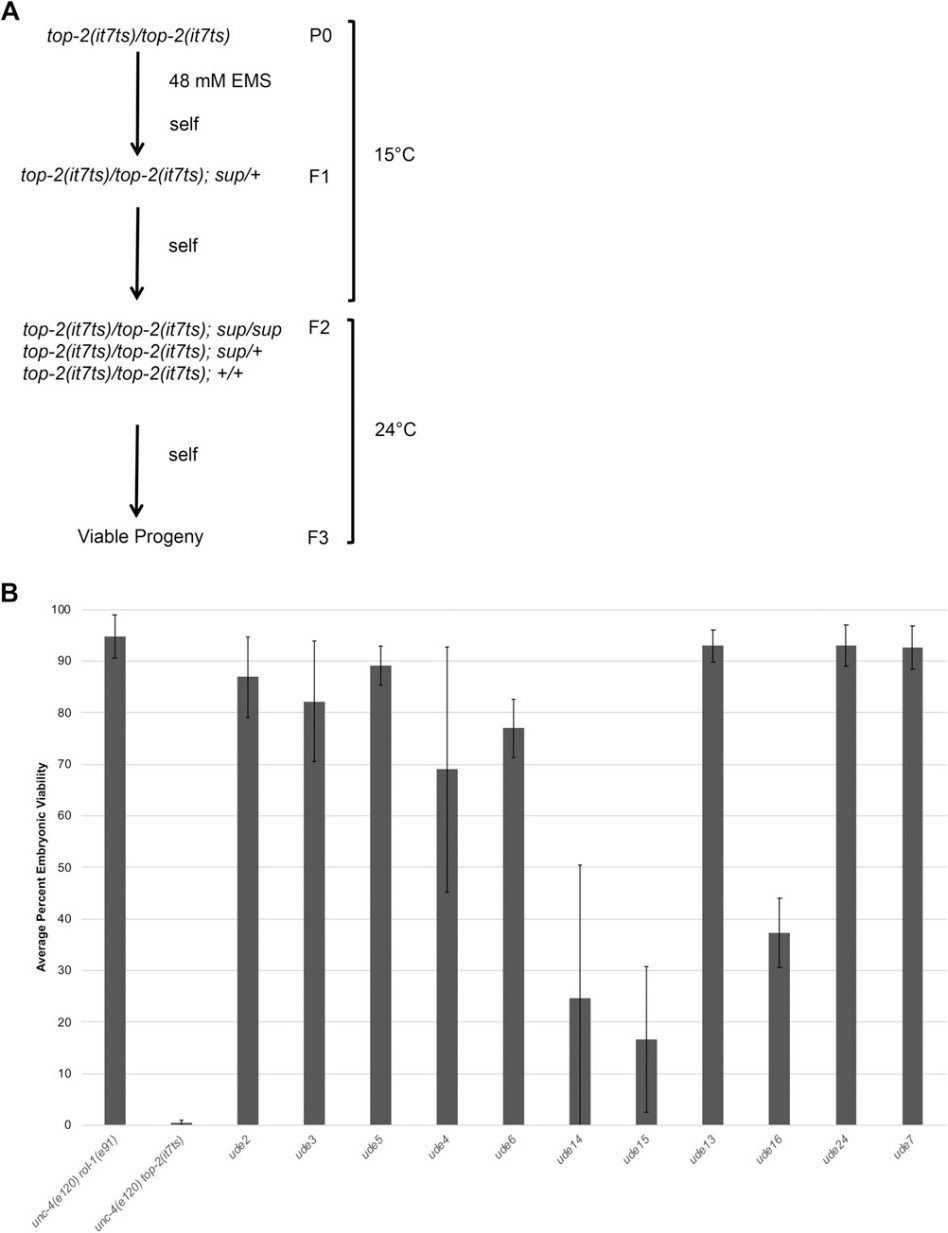
Isolation of suppressors of *top-2**(**it7ts**)* embryonic lethality. (A) Schematic of the suppressor screen to isolate mutations that suppress the temperature sensitive embryonic lethality of *top-2**(**it7ts**)*. sup = suppressor. (B) Identification of 11 suppressors of *top-2**(**it7ts**)* embryonic lethality. Graph depicts the average percent embryonic viability of at least three independent experiments at 24°C. Suppressors are labeled as *ude*, which is the allele designation assigned to the Jaramillo-Lambert laboratory. Error bars indicate standard deviation.

Previously, we identified *top-2**(**it7ts**)*, a temperature-sensitive, missense mutation in a conserved residue within the catalytic domain of TOP-2 (Jaramillo-Lambert *et al.* 2016). At 15°, *top-2**(**it7ts**)* animals produce viable progeny (94.8% hatching). In contrast, *top-2**(**it7ts**)* hermaphrodites raised at 24° produce inviable progeny (0.5% hatching) due to chromosome segregation defects during spermatogenesis [Figure 1B, (Jaramillo-Lambert *et al.* 2016)]. To identify suppressors of *top-2**(**it7ts**)* embryonic lethality, we exposed L4 *top-2**(**it7ts**)* hermaphrodites to EMS at the permissive temperature (15°), allowed the worms to grow for two generations, shifted the F2 generation to 24°, and collected rare survivors among the F3 progeny (Materials and Methods, Figure 1A). Of >80,000 haploid genomes screened, we recovered 11 independent suppressor lines.

We performed embryonic viability assays to determine the extent to which each suppressor line rescued the embryonic lethality phenotype of *top-2**(**it7ts**)*. Eight of the 11 lines showed strong suppression rescuing embryonic viability >50%. Six had nearly wild-type levels of embryonic viability with average percent viabilities of >80% (Figure 1B). One of the suppressors, *ude16*, rescued embryonic viability to 37.3%. The remaining two suppressors, *ude14* and *ude15*, rescued embryonic viability above 10% with more variability in progeny survival among individual worms (Figure 1B).

We determined the dominance or recessiveness of the suppressor mutations by crossing each suppressed line with unmutagenized *top-2**(**it7ts**)*; *him-8**(**e1489**)* males (see Materials and Methods). Of the 11 suppressor lines, ten of the suppressors were either dominant (>50% viability) or semi-dominant (10–49% viability) mutations and one suppressor mutation was recessive (<10% viability, Table 1). To differentiate between suppressors that are linked *vs.* unlinked to the *top-2* gene, we performed embryonic viability assays on Unc F2 progeny from *unc-4**(**e120**) **top-2**(**it7ts**)/**unc-4**(+) **top-2**(**it7ts**)*; *sup/+* F1 animals (see Materials and Methods). Ten of the suppressors were unlinked from *top-2* and one suppressor was linked (Table 1). Lastly, we sequenced a portion of the *top-2* gene containing codon 828 in the 11 suppressor lines to determine if any of the suppressors had mutations that reverted the original *it7ts* mutation (Arg828Cys) back to a wild-type arginine amino acid. All 11 suppressors contained the *it7ts* mutation (Table 1).

**Table 1:** Suppressor genetic analysis

Suppressor Allele	Revertant*^a^*	Dominant/ Recessive (% Embryonic Viable)*^b^*	Linked or Unlinked from *top-2(it7ts)*
*ude2*	No	Dominant (68.7%)	Unlinked
*ude3*	No	Dominant (68.3%)	Unlinked
*ude5*	No	Dominant (85.8%)	Unlinked
*ude4*	No	Semi-dominant (41%)	Unlinked
*ude6*	No	Dominant (83.3%)	Linked
*ude14*	No	Semi-dominant (25.8%)	Unlinked
*ude15*	No	Recessive (5.9%)	Unlinked
*ude13*	No	Dominant (73.9%)	Unlinked
*ude16*	No	Semi-dominant (17.9%)	Unlinked
*ude24*	No	Dominant (84.1%)	Unlinked
*ude7*	No	Dominant (84.8%)	Unlinked

a This column indicates whether the suppressor line is due to a reversion of the *top-2(it7ts)* R828C mutation back to wild type.

b Percent embryonic viability of heterozygous animals (Suppressor/+). Suppressors with >50% viability are dominant, 10–49% viability are semi-dominant, <10% viability are recessive.

ude = is the allele designation assigned to the Jaramillo-Lambert laboratory.

We used a combination of Hawaiian SNP mapping and WGS (Doitsidou *et al.* 2010; Jaramillo-Lambert *et al.* 2015), to simultaneously map and identify the suppressing mutations. We identified a single nonsynonymous protein-coding candidate mutation for the one linked suppressor, *ude6*, which is a novel missense mutation within *top-2* (Table 2).

**Table 2:** Candidate genes for the suppressors

Intragenic/Extragenic	Suppressor allele	Candidate gene	Chromosome	Mutation	RNAi of *top-2(it7ts)**^a^*
		*smd-1* (control)			0.8% (n = 8167)
Intragenic	*ude6*	*top-2*	II	D809N	NP
Extragenic	*ude2*	*tdpt-1*	I	G328E	
	*ude3*	*tdpt-1*	I	G219E	
	*ude4*	*tdpt-1*	I	G117R	
	*ude5*	*tdpt-1*	I	G270D	91.9% (n = 2253)**
	*ude24*	*tdpt-1*	I	G270D	
	*ude7*	*tdpt-1*	I	G270S	
	*ude13*	*tdpt-1*	I	A355T	
	*ude14*	*mep-1*	IV	G57D	1.0% (n = 4624)^ns^
	*ude23*	*nurf-1*	II	E1954K	1.7% (n = 4253)^ns^
		*mep-1+nurf-1*			9.2% (n = 3460)*
	*ude15*	*top-3*	III	P699S	0.9% (n = 2102)^ns^
	*ude16*	*viln-1*	I	R552C	1.0% (n = 1644)^ns^
		*nlp-38*	I	G77R	2.7% (n = 5680)^ns^
		*viln-1+nlp-38*			6.9% (n = 8068)^ns^

a Percent of viable progeny from *unc-4(e120) top-2(it7ts)* hermaphrodites fed RNAi bacteria against the gene listed in column three at 24°C.

NP = not performed.

n = the number of embryos scored.

* *P* < 0.05, one-tailed student’s T-test.

** *P* < 0.005, one-tailed student’s T-test.

^ns^ indicates the data are not significant by the one-tailed student’s T-test.

*ude* = is the allele designation assigned to the Jaramillo-Lambert laboratory.

Of the 10 unlinked suppressors, seven of the suppressors contained a single candidate mutation on LGI (Table 2). Two of these seven suppressors carried the same mutation. Although they contain the same mutation, they were isolated in two independent screens and likely carry different background mutations. Thus, we assigned them different allele designations, *ude5* and *ude24* (Tables 1 and 2). These seven suppressors, *ude2*, *ude3*, *ude4*, *ude5*, *ude7*, *ude13* and *ude24*, contain different mutant alleles within the same gene, *tdpt-1*. TDPT-1 is the *C. elegans* homolog of tyrosyl DNA phosphodiesterase 2 (Tdp2). Tdp2 is a multifunctional protein involved in DNA repair, gene transcription, and signal transduction during mitosis (Pommier *et al.* 2016) and has not previously been shown to play a role during meiosis.

The remaining three unlinked suppressors yielded more than one candidate mutation for each. *ude14* had one candidate mutation on LGII (*nurf-1**)*, and one candidate mutation on LGIV (*mep-1*, Table 2). *nurf-1* is a nucleosome remodeling complex homolog (Andersen *et al.* 2006) and *mep-1* encodes a zinc-finger protein that is part of a chromatin remodeling complex that functions in cell fate determination (Unhavaithaya *et al.* 2002). *ude16* had two candidate mutations in *viln-1* and *nlp-38* in an interval on LGI. *viln-1* is a homolog of human supervillin. The predicted gene function of *viln-1* on Wormbase (www.wormbase.org) is to bind actin filaments. *nlp-38* is predicted to encode a neuropeptide-like protein. *ude15*, contained an excess of unique mutations (20 out of 56) on LGIII, making identification of the suppressing mutation difficult. However, among the unique mutations is an allele of another topoisomerase, *top-3*, which has been shown to have roles in recombination during meiosis (Wicky *et al.* 2004). Because of this function, we focused on *top-3* as the potential suppressor candidate for *ude15* (Table 2).

### Identification of the suppressor loci

To validate the molecular lesion responsible for suppression in each suppressor line, we first tested candidates for phenocopy of suppression through RNAi knockdown. We knocked-down *tdpt-1*, *top-3*, *nlp-38*, *viln-1*, *nurf-1*, and *mep-1* in *unc-4**(**e120**) **top-2**(**it7ts**)* hermaphrodites raised at 24° and scored for the production of viable progeny. RNAi of *tdpt-1* verified the candidate gene for *ude2*, *ude3*, *ude4*, *ude5*, *ude7*, *ude13*, and *ude24*. Depletion of *tdpt-1* resulted in suppression of embryonic lethality in *unc-4**(**e120**) **top-2**(**it7ts**)* with a penetrance of 91.9% (Table 2). RNAi knockdown of *tdpt-1* in wild-type (N2) animals does not result in a decrease in embryonic viability (Figure S1A). Given that the *tdpt-1* suppressing mutations are dominant alleles, it is surprising that loss of *tdpt-1* expression through RNAi can recapitulate suppression. This indicates that the *tdpt-1* suppressing alleles are haploinsufficient and sensitive to the dose of TDPT-1 protein, thus requiring two copies of the wild-type allele to function properly. One function of Tdp2 is to remove trapped Top2-DNA covalent complexes (Top-2cc) that inhibit DNA replication and transcription (Pommier *et al.* 2014). The identification of seven mutant alleles of *tdpt-1* that suppress the *top-2**(**it7ts**)* phenotype suggests that mutant TOP-2 proteins may bind and cleave DNA during meiosis, however, they may be unable to complete their topoisomerase activity forming Top-2cc. Perhaps, when TOP-2 cannot function properly, as in the case of the *top-2**(**it7ts**)* mutant, wild-type TDPT-1 removes TOP-2 from DNA disrupting chromosome structure/chromatin function, resulting in chromosome missegregation. In this context, if TDPT-1 is rendered inactive (suppressing mutations), residual TOP-2 activity will eventually lead to chromosome segregation. RNAi knockdown of *tdpt-1* in wild-type (N2) animals is not haploinsufficient because no Top-2cc are present. This is one possible hypothesis. Future studies will elucidate the mechanism of *tdpt-1*-mediated suppression.

For *ude14* knockdown of the individual gene candidates, *mep-1* and *nurf-1*, did not suppress *unc-4**(**e120**) **top-2**(**it7ts**)* embryonic lethality (1.0% and 1.7% viable progeny, Table 2). However, previous studies found that *mep-1* and *nurf-1* interact genetically (Andersen *et al.* 2006). We used RNAi to knockdown both *mep-1* and *nurf-1* in *unc-4**(**e120**) **top-2**(**it7ts**)*. The combined knockdown of *mep-1* and *nurf-1* resulted in suppression of embryonic lethality in *unc-4**(**e120**) **top-2**(**it7ts**)* at 24° (9.2%, Table 2). Individual RNAi knockdown of either *mep-1* or *nurf-1* as well as the combined knockdown of *mep-1* and *nurf-1* in wild-type (N2) animals does not result in a decrease in embryonic viability (Figure S1A). From these data we conclude that the combination of *nurf-1* and *mep-1* mutations can suppress *top-2**(**it7ts**)*-induced embryonic lethality. In the light of this conclusion, we kept the original allele designation of *ude14* assigned to the *mep-1* G57D mutation and assigned the *nurf-1* E1954K mutation the allele designation of *ude23*. Hence, the genotype of the original suppressed line should more accurately be *top-2**(**it7ts**) **nurf-1**(**ude23**)*; *mep-1**(**ude14**)*. Both *nurf-1* and *mep-1* are involved in chromatin remodeling suggesting that a particular chromatin state is required for proper chromosome segregation. TOP-2 may be involved in providing this particular chromatin conformation.

*ude15* contains the only recessive suppressing mutation. For *ude15*, RNAi knockdown of *top-3* in the *unc-4**(**e120**) **top-2**(**it7ts**)* background failed to validate *top-3* [P699S] as a suppressor of *top-2**(**it7ts**)* embryonic lethality (0.9% viable, Table 2). Previous studies reported that RNAi knockdown of *top-3* shows various phenotypes from normal, healthy worms to animal sterility (Kim *et al.* 2000). We found that wild-type animals treated with *top-3* RNAi were both fertile and produced viable progeny (Figure S1A). This could be due to insufficient knockdown of *top-3*. We delivered the double-stranded RNA (dsRNA) to the animals using the RNAi feeding method (Timmons *et al.* 2001), while the previous study of *top-3* performed RNAi via injection of dsRNA; a more direct delivery method. RNAi by feeding is not always as potent as injection of dsRNA (Ahringer 2006). As *ude15* contained 20 unique mutations, it is highly possible that the P699S mutation identified in *top-3* is not the suppressing mutation. Additional studies will need to be performed to determine the identity of the suppressing mutation for *ude15*.

For *ude16*, the individual RNAi knockdown of *viln-1* and *nlp-38* in the *unc-4**(**e120**) **top-2**(**it7ts**)* background did not suppress embryonic lethality (1.0% and 2.7% viable respectively, Table 2). We also used RNAi to knockdown both *viln-1* and *nlp-38* in *unc-4**(**e120**) **top-2**(**it7ts**)*. The combined knockdown of *viln-1* and *nlp-38* in *unc-4**(**e120**) **top-2**(**it7ts**)* at 24° resulted in 6.9% embryonic viability, however, this was not statistically significant (*P* > 0.05, Table 2). RNAi knockdown of *viln-1* and *nlp-38*, as well as the combined knockdown of *viln-1* and *nlp-38* in wild-type (N2) animals did not decrease embryonic viability (Figure S1A). From these data, RNAi in the *unc-4**(**e120**) **top-2**(**it7ts**)* background did not positively identify either *viln-1* [R552C] or *nlp-38* [G77R] as the suppressing mutations. However, as the *ude16* suppressing mutation is dominant, we reasoned that it may also be a gain-of-function mutation. To that end we performed RNAi in the suppressed line to determine if gene knockdown would reverse suppression. Using this approach, we found that RNAi of *viln-1*, *nlp-38*, and the *viln-1**/**nlp-38* combination did not reverse suppression (Table S2). This suggests that WGS has not identified the correct suppressing mutations or that RNAi knockdown of these genes is not efficient in eliciting the suppressing phenotype. Additional studies will need to be performed to determine the identity of the suppressing mutations of the *ude16* suppressor.

**Figure 2:**
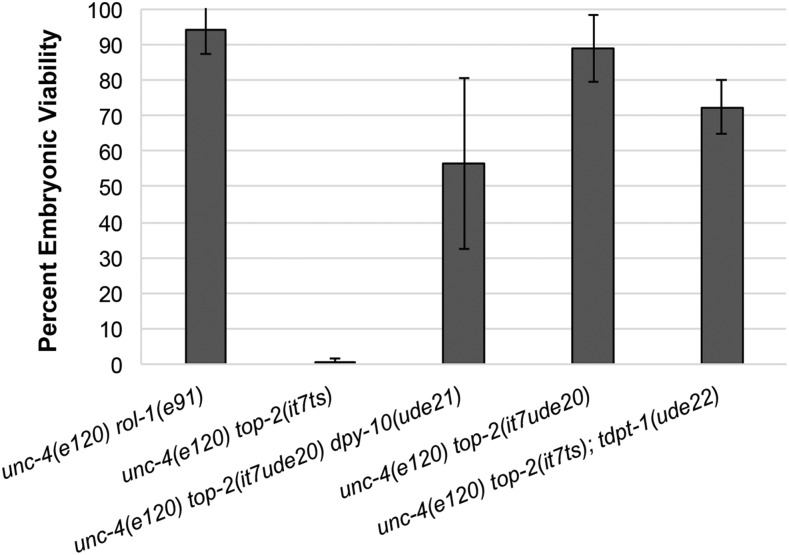
Single point mutations suppress *top-2**(**it7ts**)* embryonic lethality. Percent embryonic viability at 24°C for control lines [*unc-4**(**e120**) **rol-1**(**e91**)* and *unc-4**(**e120**) **top-2**(**it7ts**)*] and the CRISPR/Cas9 mediated recreation of the suppressor lines [*unc-4**(**e120**) **top-2**(**it7**ude20**) **dpy-10**(ude21)*, *unc-4**(**e120**) **top-2**(**it7**ude20**)* (*ude6* D809N recreated lines), and *unc-4**(**e120**) **top-2**(**it7ts**)*; *tdpt-1**(**ude22**)* (*ude5* G270D recreate)]. Error bars indicate standard deviation of at least three independent experiments. The progeny of at least 30 hermaphrodites were scored for each genotype.

As further validation that mutations in *tdpt-1* result in *top-2**(**it7ts**)* suppression of embryonic lethality, we used CRISPR/Cas9 genome editing to recreate one of the identified *tdpt-1* mutations. We identified three suppressors that resulted in missense alleles at amino acid 270 of TDPT-1, suggesting that this site is very important for TDPT-1 function. For this reason, we recreated the *tdpt-1**(**ude5**)* and *tdpt-1**(**ude24**)* G270D missense mutation in the *unc-4**(**e120**) **top-2**(**it7ts**)* strain originally used for the suppressor screen. We generated one line and assigned it the allele designation *ude22*. While *unc-4**(**e120**) **rol-1**(**e91**)* WT control viability was 94.2%, embryonic viability of *unc-4**(**e120**) **top-2**(**it7ts**)* was 0.6% at 24° (Figure 2). Embryonic viability of the *tdpt-1* G270D mutation [*unc-4**(**e120**) **top-2**(**it7ts**)*; *tdpt-1**(**ude22**)*] was 72.2% (Figure 2). We conclude, from this data along with the RNAi data, that mutations in *tdpt-1* can suppress *top-2**(**it7ts**)*-induced embryonic lethality.

We also used CRISPR/Cas9 genome editing to validate the one intragenic suppressor through recreation of the *top-2**(**ude6**)* D809N allele in the *unc-4**(**e120**) **top-2**(**it7ts**)* line. We recovered two independent, edited lines, one linked and one unlinked from *dpy-10*, and assigned them the allele designation *ude20*. Embryonic viability of *unc-4**(**e120**) **top-2**(**it7ts**)* was 0.6% (at 24°) while embryonic viability of the recreated lines were 56.5% and 88.7% respectively [*unc-4**(**e120**) **rol-1**(**e91**)* control viability was 94.2%, Figure 2]. These data confirm that the *ude6* allele (D809N) of *top-2* suppresses the embryonic lethality caused by the R828C mutation of *top-2**(**it7ts**)*. Interestingly, this suppressing mutation also falls within the topoisomerase domain perhaps compensating for structural changes caused by the R828C mutation in *top-2**(**it7ts**)*. Animals bearing a CRISPR recreated version of *top-2**(**ude6**)* in a wild-type (N2) background were healthy and produced viable progeny at 24° (Figure S1B). Future studies will determine the structural and functional roles of the R828 and D809 amino acids in TOP-2 function during meiosis.

### Characterization of the suppressors

**Figure 3:**
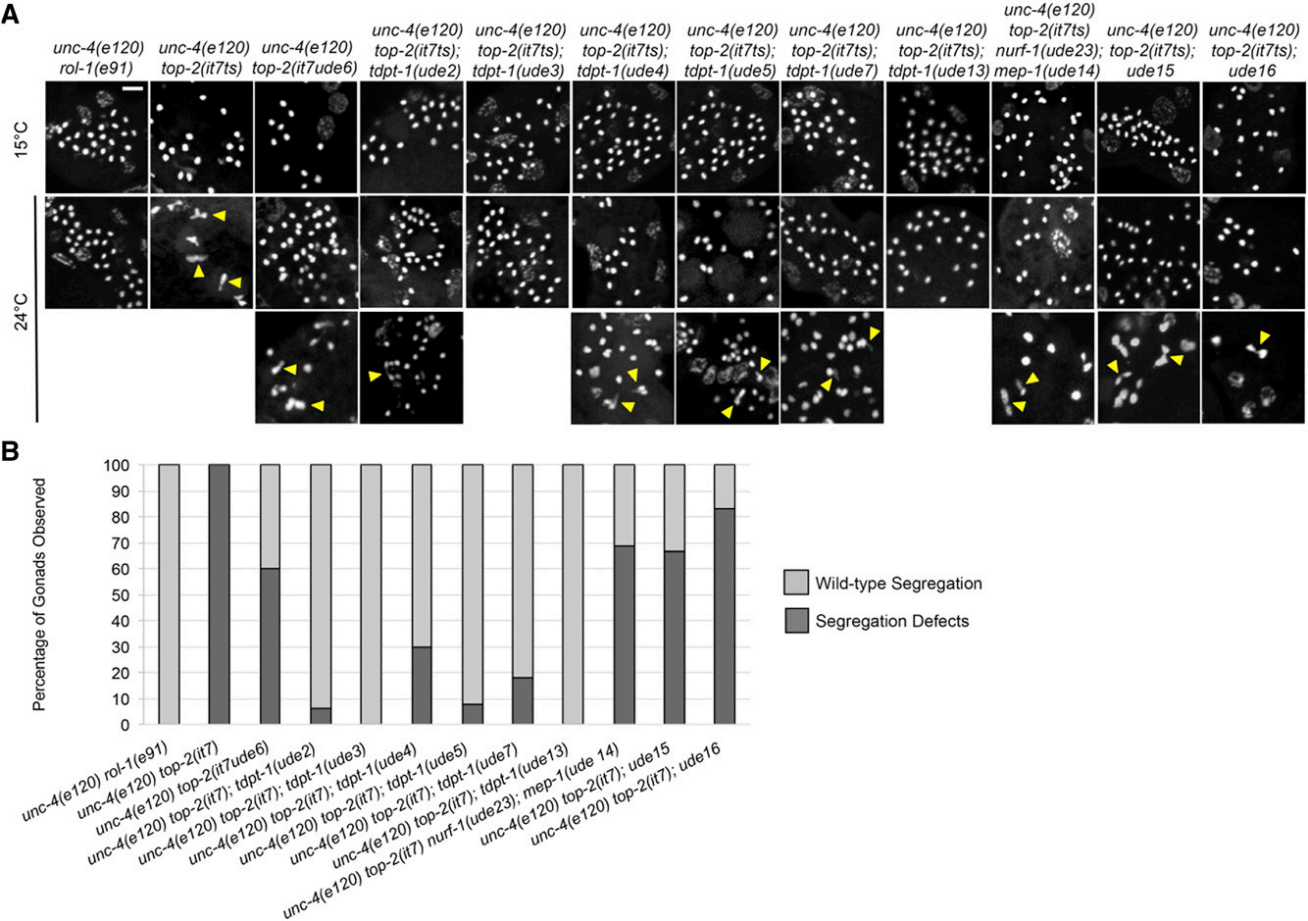
Suppressors ameliorate the chromosome segregation defects of *top-2**(**it7ts**)*. (A) Chromosomes in post-meiotic sperm of hermaphrodite young adults (24 h post L4 at 15°C and 24°C) of control lines [*unc-4**(**e120**) **rol-1**(**e91**)* and *unc-4**(**e120**) **top-2**(**it7ts**)*] and the suppressor lines. Chromosome segregation defects were assessed via confocal imaging of DAPI stained dissected gonads and spermathecae. Arrowheads in yellow indicate chromatin bridges. At least 30 gonads were examined for each genotype. Scale bar = 5 μm. (B) Quantification of chromosome segregation defects at 24°C from (A). Dark gray bar= percent of gonads with at least one chromosome segregation defect. Light gray bar= percent of gonads with no chromosome segregation defects (wild-type chromosome segregation). At least 30 gonads were examined for each genotype.

All 11 of the suppressors rescue the embryonic lethality of *top-2**(**it7ts**)* with varying degrees of penetrance (Figure 1B). To determine whether the suppressors can rescue other *top-2**(**it7ts**)* phenotypes, we examined post-meiotic sperm in the spermathecae of suppressor hermaphrodites for rescue of chromosome segregation defects by DAPI staining after 16 h incubation at 24° (shift at L3/L4 larval stage). Post-meiotic sperm of wild-type hermaphrodites [*unc-4**(**e120**) **rol-1**(**e91**)*] have a round and compact morphology (Shakes *et al.* 2009) while *unc-4**(**e120**) **top-2**(**it7ts**)* post-meiotic sperm have abnormal chromosome morphology with chromatin bridges [Figure 3A and 3B, (Jaramillo-Lambert *et al.* 2016)]. We found that all 11 suppressors can rescue chromosome segregation defects with varying penetrance (Figure 3A and 3B). *tdpt-1**(**ude3**)* and *tdpt-1**(**ude13**)* suppressor lines were the most similar to wild type with 0% of germ lines displaying abnormal chromosome morphology (Figure 3B). *ude15*, *ude16*, and the double mutant *mep-1**(**ude14**)*; *nurf-1**(**ude23**)* were the least efficient suppressors with 66.7%, 83.3%, and 68.8% of gonads with abnormal chromosome structures. Interestingly, the intragenic suppressor, *ude6*, had one of the highest percent embryonic viabilities of the suppressors (77% viable, Figure 1B), but had one of the highest percent of gonads with at least one chromatin bridge (60% of gonads with chromosome structure defects, Figure 3A and 3B).

**Figure 4:**
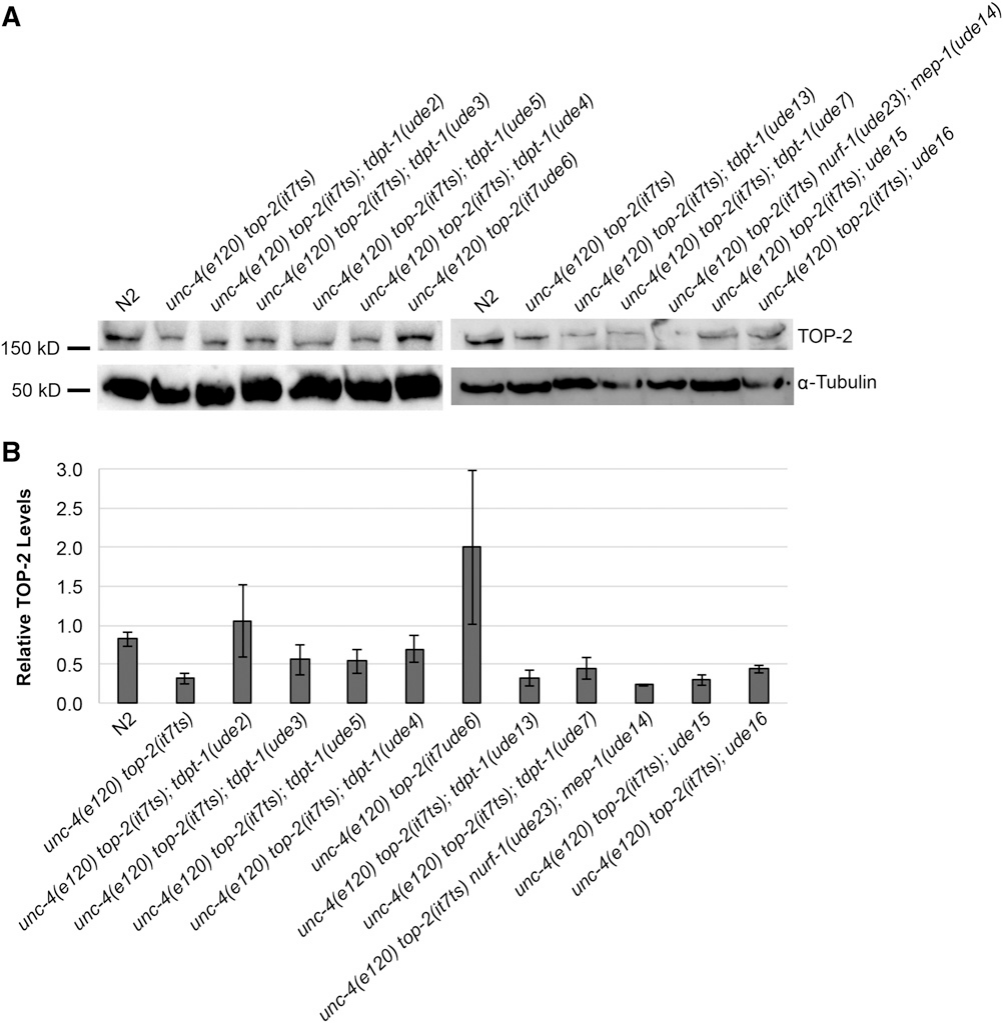
Some suppressors restore TOP-2 protein levels. (A) Western blot analysis of TOP-2 protein levels in the suppressors grown at 24°C in relation to *unc-4**(**e120**)**rol-1**(**e91**)* control and the *top-2**(**it7ts**)* mutant. (B) Quantification of TOP-2 protein levels compared to ⍺-Tubulin loading controls. Data show the average of at least three experiments. Error bars represent SEM of at least three individual experiments.

Next, we assessed TOP-2 protein levels in the suppressor lines compared to wild type (N2) and *unc-4**(**e120**) **top-2**(**it7ts**)* mutant lines. Although, we previously demonstrated that the primary defect of *top-2**(**it7ts**)* is a failure to localize to chromosomes, we also showed that TOP-2 protein levels via quantification of pixel intensities indicated that TOP-2 protein is reduced in late meiotic prophase of *top-2* mutant germ lines [*top-2**(**av77**)*::*3xflag*, a CRISPR recreation of *top-2**(**it7ts**)* (Jaramillo-Lambert *et al.* 2016)]. To more directly assess TOP-2 protein levels, we generated a *C. elegans*-specific anti-TOP-2 polyclonal antibody and examined protein levels by Western blotting. TOP-2 protein levels were reduced after RNAi-mediated knockdown of *top-2* and in *top-2**(**ok1930*Δ*)* confirming antibody specificity (Figure S2). As expected, at 24°, TOP-2 protein levels were reduced in *unc-4**(**e120**) **top-2**(**it7ts**)* whole worm lysates compared to wild-type control (N2, Figure 4A and 4B). Although several of the suppressors did not restore TOP-2 protein levels [*ude13*, *ude14*; *ude23*, and *ude15*], seven of the suppressors, *ude2*, *ude3*, *ude4*, *ude5*, *ude6*, *ude7*, and *ude16* had TOP-2 protein levels that were intermediate between *unc-4**(**e120**) **top-2**(**it7ts**)* and wild type or restored to wild-type TOP-2 protein levels (Figure 4A and 4B). Quantification by densitometry, indicates that TOP-2 protein levels are increased the most in *ude6*. As *ude6* is the *top-2* intragenic suppressor it is possible that the D809N suppressing mutation is creating some sort of structural compensation for the original *top-2**(**it7ts**)* [R828C] mutation. Even though the primary defect of *top-2**(**it7ts**)* is a failure to localize to meiotic chromosomes, this study demonstrates that *top-2**(**it7ts**)* also has a defect in TOP-2 protein stability and that some of the suppressors can restore TOP-2 protein stability. Future studies will examine the mechanisms of suppression for the validated suppressors.

## Conclusion

In summary, we identified 11 genetic suppressors of *top-2**(**it7ts**)* embryonic lethality. We identified the molecular lesion responsible for suppression of nine of the suppressor candidates via whole-genome sequencing and single-nucleotide polymorphism mapping and verified either through RNAi phenocopy or through CRISPR/Cas9 recreation of the suppressing mutation. The genes identified in this screen comprise different functional classes including chromatin remodeling, DNA repair, and protein structure/stability. The intragenic mutation, *ude6*, most likely helps restore protein structure and stability as TOP-2 protein levels are decreased in the *top-2**(**it7ts**)* mutant line and restored in the *top-2**(**it7**ude6**)* suppressor line (Figure 4). MEP-1 and NURF-1 are both involved in chromatin remodeling and interact genetically. TDPT-1 is involved in the removal of TOP-2 protein complexes and DNA repair. How these proteins interact with TOP-2 in the context of homologous chromosome segregation during meiosis is unknown. Future studies will help determine the mechanism of suppression of these different mutations and will elucidate the genetic pathways in which these genes interact with *top-2* during meiosis.
